# Efficacy of steroid therapy in the acute stage of anti-NMDAR and anti-MOG antibody overlapping encephalitis: a case report and literature review

**DOI:** 10.3389/fimmu.2024.1392992

**Published:** 2024-06-04

**Authors:** Hikari Kondo, Yuko Takeuchi, Junichi Niwa, Kenji Yoshida, Naoaki Takemura, Sachiko Hosoyama, Tomotsugu Kaga, Kimihiko Kaneko, Naoki Mabuchi

**Affiliations:** ^1^ Department of Neurology, Nagoya Ekisaikai Hospital, Nagoya, Japan; ^2^ Department of Neurology, Masuko Memorial Hospital, Nagoya, Japan; ^3^ Department of Neurology, Stroke Center, Aichi Medical University, Nagakute, Japan; ^4^ Department of Neurology, Tohoku University, Sendai, Japan

**Keywords:** anti-NMDAR encephalitis, autoimmune encephalitis, myelin oligodendrocyte glycoprotein (MOG), N-methyl-D-aspartate receptor (NMDAR), overlapping encephalitis, steroid

## Abstract

**Background:**

Recently, cases of overlapping encephalitis caused by anti-N-methyl-D-aspartate receptor (anti-NMDAR) and anti-myelin oligodendrocyte glycoprotein (MOG) antibodies have been reported, and their clinical characteristics are gradually becoming clear. Acute-phase treatment typically involves the use of steroids, and although some studies have suggested that steroids can be effective, the extent of their efficacy has not yet been fully explored.

**Case presentation:**

We present the case of a 25-year-old man with anti-NMDAR and anti-MOG antibody overlapping encephalitis who showed considerable improvement after steroid treatment. To gain a deeper understanding of the efficacy of steroids in managing this condition, we conducted a literature review of cases of anti-NMDAR and anti-MOG antibody double-positive encephalitis that were treated with steroids during the acute phase. Thirteen cases were analyzed, including a new case diagnosed at our hospital. All patients showed improvement after receiving steroid treatment in the acute phase. Ten patients did not have any sequelae, and nine of them showed a rapid or major response during the acute phase. In contrast, three patients experienced sequelae (mild cognitive decline, visual impairment, and memory impairment, respectively), with their response to steroids in the acute phase being slow or limited. Relapses occurred in five patients, in one patient during steroid tapering, and in another two patients after cessation of steroids.

**Conclusion:**

Steroid therapy can be effective in the acute stage of anti-NMDAR and anti-MOG antibody overlapping encephalitis. A positive prognosis may be expected in patients who experience substantial improvement with steroid therapy during the acute phase.

## 1 Introduction

Anti-N-methyl-D-aspartate receptor (anti-NMDAR) encephalitis is an autoimmune disorder that causes brain inflammation. The initial report of anti-NMDAR encephalitis involved 12 young women, of whom 11 (91.7%) had ovarian teratomas (1). However, a subsequent multicenter study found that 35.9% of patients had ovarian teratomas, and it is now recognized that the disease can occur in individuals of any age, regardless of sex or the presence of tumors (2). The primary symptoms of anti-NMDAR encephalitis include psychiatric disorders, seizures, impaired consciousness, and involuntary movements (3). Brain imaging often reveals no obvious abnormalities. The treatment of anti-NMDAR encephalitis typically involves intravenous methylprednisolone (IVMP), intravenous immunoglobulins (IVIG), and plasma exchange (PE); however, only half of these cases show improvement with treatment (2). The acute stage of the disease often requires a prolonged hospital stay of 3–4 months, followed by rehabilitation (4). The condition generally follows a monophasic course with a 2-year relapse rate of approximately 12% (2).

Myelin oligodendrocyte glycoprotein (MOG) is a component of the myelin sheath of the central nervous system. MOG antibody-associated disease (MOGAD) is a newly recognized clinical condition that results in the inflammation and destruction of myelin in the central nervous system (5). Most patients with MOGAD respond well to steroid treatment (6); however, approximately 45% of these patients experience recurrence of symptoms within 2 years after disease onset (7).

Over the past few years, anti-NMDAR encephalitis has been reported to be associated with MOG antibodies. Some researchers have considered this condition as another type of autoimmune encephalitis, distinct from anti-NMDAR encephalitis or MOGAD alone. Since Titulaer et al. first reported an overlapping syndrome of anti-NMDAR and anti-MOG antibodies (8), a growing number of cases have been documented, and their clinical characteristics have become more apparent. Previous reviews have identified several features of this condition, including its higher prevalence in young men (9–13); symptoms similar to anti-NMDAR encephalitis, such as psychiatric symptoms and seizures (10, 12, 13); complex imaging findings with features of both anti-NMDAR encephalitis and MOGAD (10, 12, 13); and a generally favorable functional prognosis (9–13), despite the tendency for recurrence (9–12). Initial treatment often involves steroids. Although some studies have pointed out their effectiveness, previous studies have not thoroughly examined the response to steroid therapy.

This study aimed to report about a patient with encephalitis showing both anti-NMDAR and anti-MOG antibodies who responded favorably to steroids and to review and discuss existing literature on the effectiveness of steroid therapy during the early stages of this condition.

## 2 Case presentation

**Figure 1:**
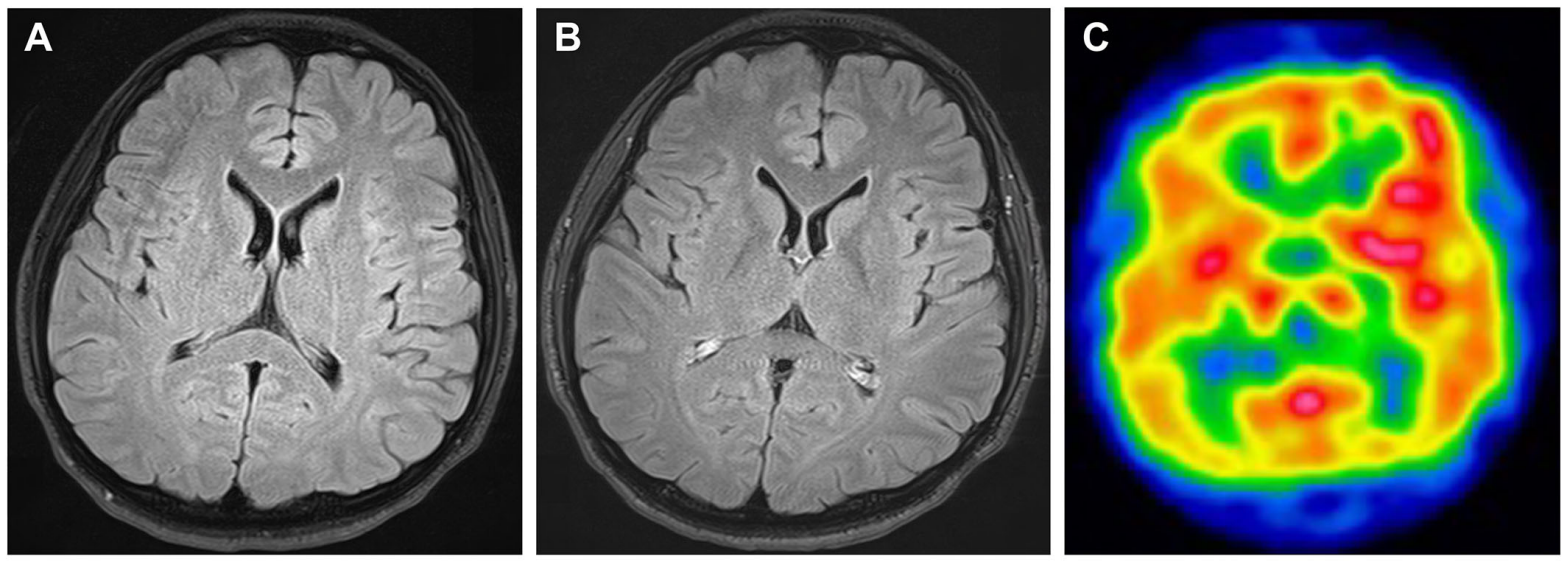
Brain images taken prior to immunotherapy. No abnormalities are obvious in fluid-attenuation inversion recovery (FLAIR) magnetic resonance imaging **(A)** or contrast-enhanced FLAIR imaging **(B)**. Brain single-photon emission computed tomography displayed increased 99mTc-ECD uptake in the left frontotemporal region **(C)**.

A previously healthy 25-year-old right-handed man complained of fever and left-sided headache that worsened over time. Seven days later, he was found walking around the room and talking to himself, followed by generalized tonic–clonic seizures that lasted approximately 1–2 minutes, with loss of consciousness. The patient denied any history of trauma, infection, alcoholism, or drug abuse. The patient’s family genetic history was unremarkable. During the neurological examination, he was found to have mild impairment of consciousness (Glasgow Coma Scale score: E4V4M5), aphasia, acalculia, oral dyskinesia, myoclonus of the right upper extremity, and right-hand clumsiness. On further examination, no notable cranial nerve, sensory testing, tendon reflex, or meningeal sign abnormalities were detected. Brain magnetic resonance imaging (MRI) findings were normal; however, brain single-photon emission computed tomography revealed increased technetium-99m ethyl cysteinate dimer (99mTc-ECD) uptake in the left frontotemporal region (**Figure 1**). Electroencephalography showed intermittent frontal rhythmic delta activity, which was more prominent in the left hemisphere. The pressure of the cerebrospinal fluid (CSF) was 130 mmH_2_O. CSF examination revealed leukocytosis (165/μL; 80% mononuclear), mildly increased protein (48 mg/dL, normal range < 40 mg/dL), and normal glucose (67 mg/dL). The immunoglobulin G (IgG) index was 0.65, and 10 CSF restricted oligoclonal bands were detected. Myelin basic protein was negative. Cytology results were negative for tumor cells, and blood tests for tumor markers were negative. Computed tomography (CT) scans of the chest, abdomen and pelvis did not reveal any tumors. All tests for bacteria, fungi, acid-fast bacilli, viral infections, and systemic autoimmune markers were negative.

Initially, the patient was suspected of having nonconvulsive status epilepticus associated with viral encephalitis and was administered intravenous acyclovir and antiseizure drugs. Despite receiving this treatment, his level of consciousness continued to decline, and his involuntary movements and abnormal behaviors worsened. On the ninth day after admission, he was barely able to speak. Since the patient did not respond to acyclovir and antiseizure medications, autoimmune encephalitis was suspected. The patient’s CSF and serum, which had been cryopreserved at the time of admission, were sent to the Department of Animal Model Development, Brain Research Institute, Niigata University, Japan. On the tenth day after admission, the patient was administered IVMP at 1000 mg/day for 5 days empirically prior to the results. The day after administration, his level of consciousness improved significantly, allowing him to engage in simple conversations and recover from right-hand clumsiness and psychobehavioral abnormalities. After 3–4 days of treatment, he underwent cognitive function tests and scored 24/30 on the Mini-Mental State Examination and 14/18 on the Frontal Assessment Battery. After the administration of a second IVMP regimen (1000 mg/day for 3 days), his involuntary movements and mild cognitive decline were relieved, resulting in the full resolution of his symptoms. The patient was discharged on the 35th day and returned to work.

A fixed cell-based assay returned positive results for anti-NMDAR antibodies in the CSF, whereas the serum was negative. He also tested positive for anti-MOG antibodies in both the CSF and serum. Test results were negative for antibodies against contactin-associated protein-like 2 (CASPR2), amino-3-hydroxy-5-methyl-isoxazolepropionic acid receptor (AMPAR), leucine-rich glioma-inactivated 1 (LGI1), dipeptidyl-peptidase-like protein 6 (DPPX), and gamma-aminobutyric acid B (GABAB) receptor. Stored serum samples collected on admission tested negative for anti-aquaporin-4 (AQP4) antibodies by enzyme-linked immunosorbent assay using commercially available kits (RSR Limited, Cardiff, UK). Additional tests performed by a commercially available fixed cell-based assay indirect immune-fluorescence method (Euroimmun, Lübeck, Germany) revealed that the patient had a CSF anti-NMDAR antibody titer of 1:20 (positivity ≥1:1). The patient’s CSF and serum anti-MOG antibodies were measured at the Department of Neurology, Tohoku University, Japan, using a cell-based assay with full-length human MOG-transfected human embryonic kidney cells 293 using goat anti-human Fc-specific IgG as a secondary antibody (Pierce Biotechnology, Rockford, USA), revealing both titers of 1:1024 (positivity ≥1:128 in serum, ≥1:1 in CSF). The cryopreserved CSF sample obtained after the first course of IVMP (on the 17th day after admission) showed a decreased anti-MOG antibody titer of 1:8.

**Figure 2:**
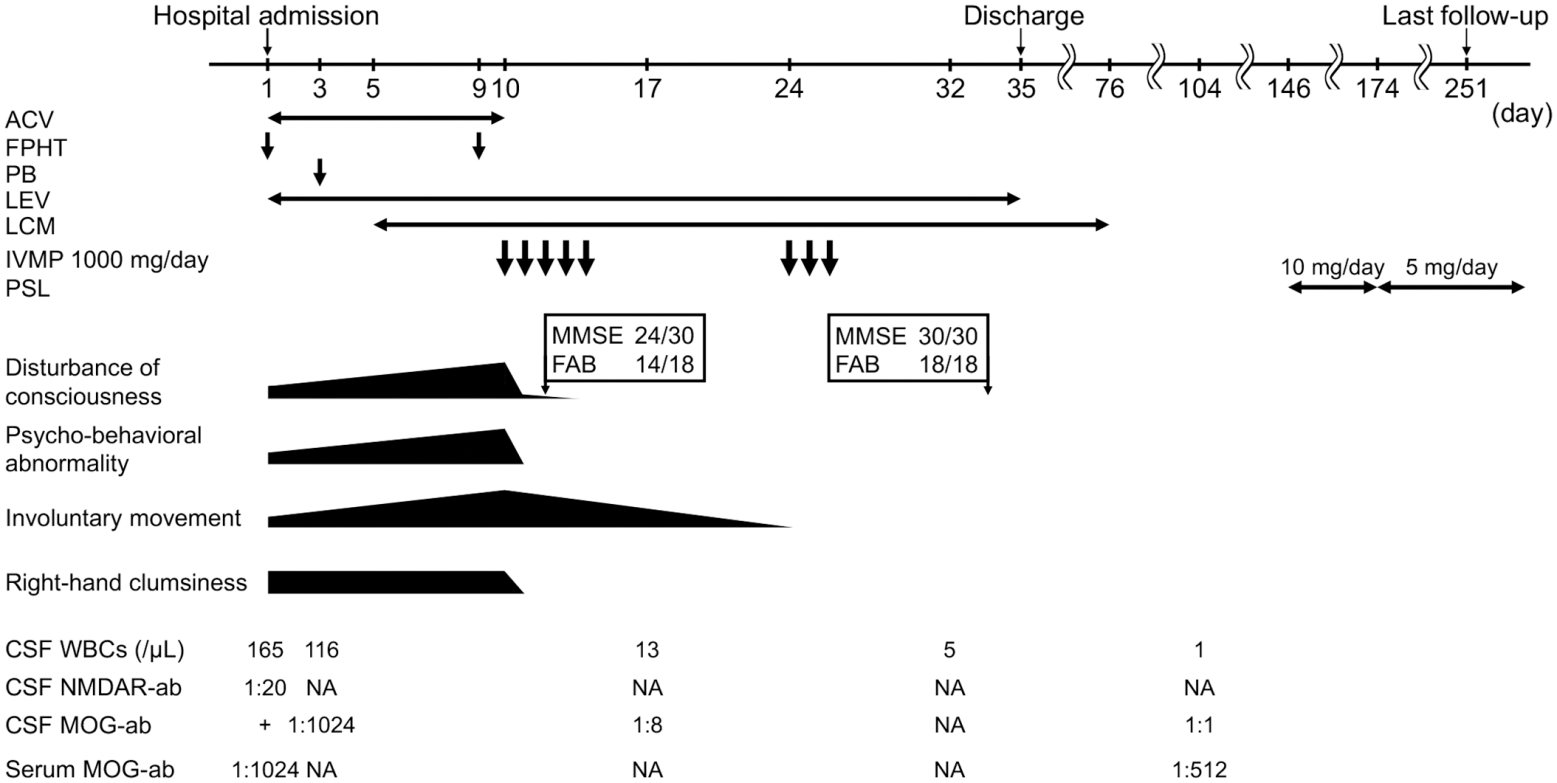
Timeline of clinical manifestations, treatment, and CSF and serum findings in our patient. ACV, acyclovir; FPHT, fosphenytoin; PB, phenobarbital; LEV, levetiracetam; LCM, lacosamide; IVMP, intravenous methylprednisolone; PSL, prednisolone; CSF, cerebrospinal fluid; WBC, white blood cell; NMDAR-ab, anti-N-methyl-D-aspartate receptor antibody; MOG-ab, anti-Myelin oligodendrocyte glycoprotein antibody; NA, not available; MMSE, Mini-Mental State Examination; FAB, Frontal Assessment Battery.

A follow-up examination 2 months later revealed that anti-MOG antibody titer in his CSF had decreased to 1:1, whereas anti-MOG antibody levels in his serum remained high (titer 1:512). Therefore, oral prednisolone (PSL) was initiated at a dose of 10 mg/day to prevent recurrence. However, due to the development of depressive symptoms, which was suspected to be a side effect of PSL, the dose was subsequently reduced to 5 mg, and the symptoms resolved. The patient achieved complete remission with no neurological defects, side effects, relapses, or infectious events at the seven-month follow-up and continued to receive oral PSL. The timeline of the patient’s clinical presentation, examination findings, and interventions is shown in **Figure 2**.

## 3 Literature search

**Figure 3:**
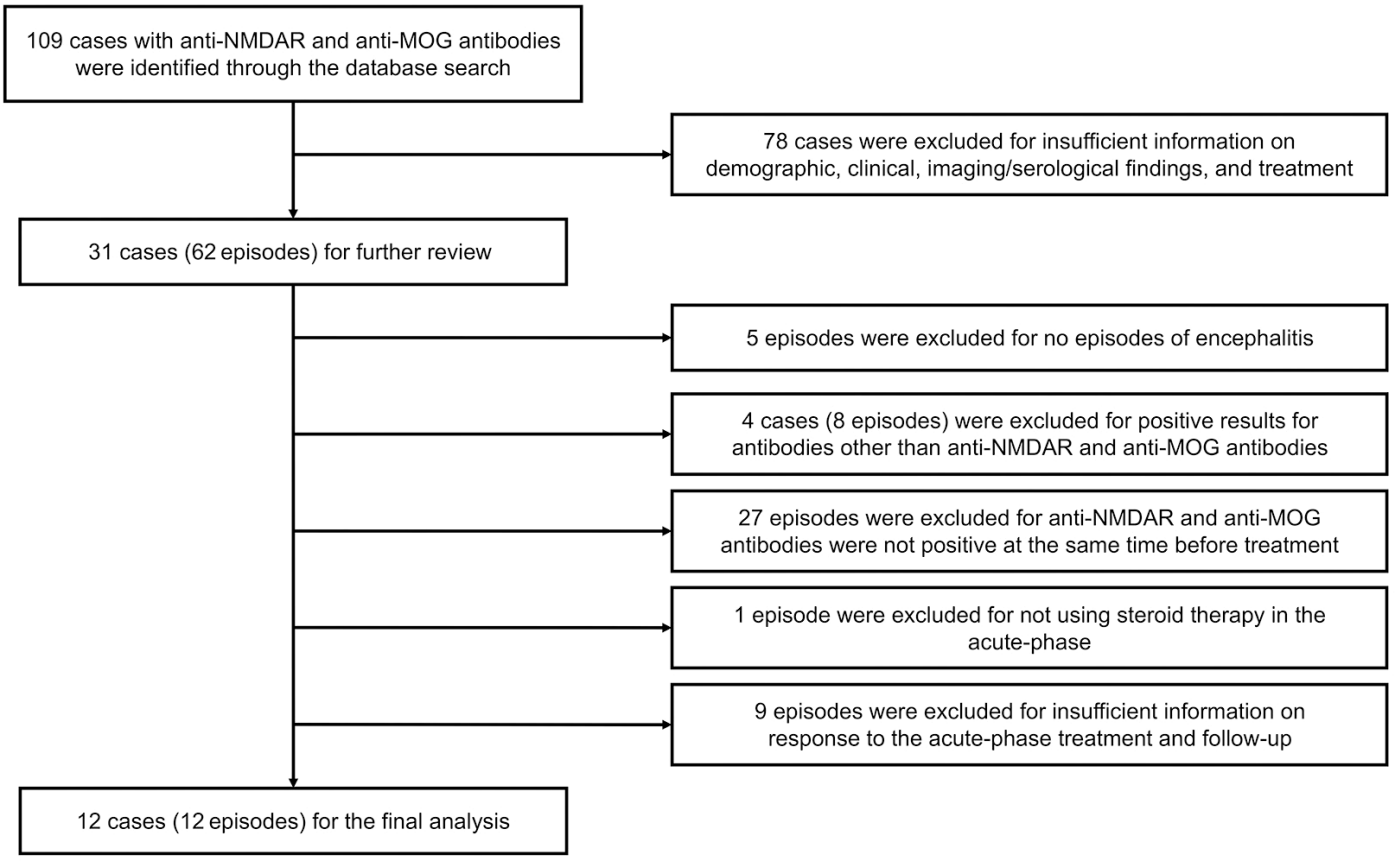
Study flowchart.

The literature search strategy followed the guidelines provided by the Preferred Reporting Items for Systematic Reviews and Meta-Analyses (PRISMA). The search was conducted in the PubMed database up to December 4, 2023, using the terms “NMDAR” and “MOG,” and the reference lists of the retrieved papers were also screened to include all previously reported cases. To provide a comprehensive overview of patient characteristics, patients with incomplete information on demographic, clinical, and imaging/serological findings and treatment were excluded. The following cases and episodes were also excluded: (i) episodes without encephalitis; (ii) cases with positive tests for antibodies other than anti-NMDAR and anti-MOG antibodies; (iii) episodes in which pretreatment specimens were not simultaneously positive for anti-NMDAR and anti-MOG antibodies; (iv) episodes in which steroids were not used in the acute phase; and (v) episodes lacking information on response to acute-phase treatment and follow-up. One newly diagnosed patient at our hospital was also included in this study. The flowchart of the study is shown in **Figure 3**.

The search in PubMed identified 109 patients. After further assessment, 13 patients (13 episodes), including the present patient, were selected for further review. Information on enrolled patients is presented in **Table 1** (9, 14–24). Among the 13 patients, 10 were male (76.9%). The age of onset ranged from 6 to 54 years, with a median of 25 years. The main clinical manifestations of this overlapping encephalitis were headache and psychiatric symptoms (53.8%); cognitive impairment, seizure, and paralysis (46.2%); fever (38.5%); impaired consciousness and sensory disturbance (30.8%); and involuntary movement and sleep disorder (23.1%). Only one patient had a complication of optic neuritis. MRI was performed for all episodes before starting acute-stage immunotherapy. MRI revealed abnormalities in the cerebral cortex (46.2%), subcortical white matter, meninges, and pons (23.1%), and thalamus and midbrain (15.4%). Three (23.1%) patients had no obvious brain lesions. One patient with optic neuritis demonstrated contrasting effects in bilateral optic nerves. Only one case was associated with ovarian teratomas, and surgical ovarian removal was performed during the acute phase; the remaining cases showed no signs of tumors.

**Table 1:** Overview of cases included in this study.

Case (Ref)	Age (years)/Sex	Symptoms	MRI lesions	Tumor	NMDAR-ab		MOG-ab		Acute-phase immunotherapy (timing)	Treatment response	Long-term immunotherapy	Relapse	Sequelae	Interval to the last F/U or relapse (mo)
					CSF	Serum	CSF	Serum						
1 (14)	9/F	Fever, impaired consciousness, cognitive impairment, paralysis, dysphagia	Cerebral cortex	No	+	+	NA	+	IVMP, IVIG (NA)	Symptom free at discharge	PSL	Yes	No	5
2 (15)	54/M	Impaired consciousness, psycho-behavioral symptoms, sleep disorder, ataxia, dizziness	Parenchyma surrounding the third ventricle, midbrain, cerebellum	No	1:32	NA	NA	1:320	IVMP, IVIG, RTX (NA)	Discharged home on the 21st day, with mild gait disturbance	PSL	No	No	2
3 (16)	6/M	Psycho-behavioral symptoms, sleep disorder, speech disorder, gait disturbance	Normal	NA	+	NA	NA	1:320	IVMP (NA)	Improved on the third day of the treatment, discharged with full recovery	No	No	No	3
4 (17)	22/F	Headache, fever, seizure, cognitive impairment, psychiatric symptoms, sensory disturbance	Cerebral cortex, meninges	Yes	+	−	Very weak	Very weak	IVMP, IVIG, PE (NA)	Resolved completely, discharged on the 23rd day	No	Yes	No	3
5 (18)	31/M	Headache, cognitive impairment, psychiatric symptoms, involuntary movement, paralysis, sensory disturbance, weight loss	Deep white matter, internal capsule, thalamus, pons	No	+	−	NA	+	IVMP, PE (third day of admission)	Some of the symptoms improved after treatment	RTX	No	Mild cognitive impairment	6
6 (19)	37/M	Headache, seizure, paralysis	Cerebral cortex, meninges	NA	1:10	1:10	1:10	1:10	PSL (NA)	Resolved completely during treatment	No	No	No	24
7 (9)	19/M	Impaired consciousness, paralysis, sensory disturbance	Subcortical white matter, basal ganglia, thalamus, pons	NA	+	−	−	+	IVMP, IVIG (NA)	Recovered quickly from lethargy	PSL	Yes	No	2
8 (20)	36/M	Headache, speech disorder, sensory disturbance	Midbrain, pons	NA	−	1:10	+	+	DEX (NA)	Relieved quickly, discharged on the 17th day without any sequela	PSL	Yes	No	14
9 (21)	28/M	Headache, fever, seizure, visual impairment, dizziness	Cerebral cortex, subcortical white matter, optic nerve	No	1:10	NA	1:32	1:10	IVMP (59th day after onset)	Some of the symptoms disappeared two months after discharge	PSL, MMF	No	Visual impairment	6
10 (22)	30/M	Headache, fever, seizure, cognitive impairment, psycho-behavioral symptoms	Cerebral cortex, meninges	NA	1:10	1:10	1:100	1:1000	IVMP, IVIG (NA)	Improved gradually	PSL, CPA	No	No	24
11 (23)	16/M	Cognitive impairment, psychiatric symptoms, involuntary movement, paralysis	Normal	NA	1:10	1:10	−	1:32	IVMP, IVIG, CPA (NA)	Mild remission, relieved gradually	PSL	Yes	Memory impairment	1
12 (24)	20/F	Seizure, sleep disorder	Cerebral cortex, subcortical white matter	No	1:10	NA	NA	1:32	IVMP (NA)	Resolved after a month	PSL	No	No	6
13 (Our case)	25/M	Headache, fever, seizure, impaired consciousness, cognitive impairment, psycho-behavioral symptoms, involuntary movement, paralysis	Normal	No	1:20	−	1:1024	1:1024	IVMP (10th day of admission)	Improved significantly, some of the symptoms disappeared the day after treatment	PSL	No	No	7

Ref, reference; M, male; F, female; NA, not available; NMDAR-ab, anti-N-methyl-D-aspartate receptor antibody; MOG-ab, anti-myelin oligodendrocyte glycoprotein antibody; IVMP, intravenous methylprednisolone; IVIG, intravenous immunoglobulins; RTX, rituximab; PE, plasma exchange; PSL, prednisolone; DEX, dexamethasone; CPA, cyclophosphamide; MMF, mycophenolate mofetil; F/U, follow-up; mo, months. –, + mean negative, positive.

The presence of anti-NMDAR antibodies in the CSF was confirmed in all cases, with 12 positive results and titers ranging from 1:10 to 1:32. Serum anti-NMDAR antibodies were tested in nine cases, returning positive results in five cases, with a titer of 1:10. The presence of anti-MOG antibodies was tested in the CSF of eight cases, with six returning positive results (titers ranging from very weak to 1:1024). In all cases, serum anti-MOG antibodies were tested, and all results were positive (titers ranging from very weak to 1:1024).

In the identified literature, all data were available for acute-phase immunotherapy, treatment responses, and long-term immunotherapy. Follow-up data were also available for all episodes for a median period of 6 months (ranging from 1 to 24 months). Steroids were used in all episodes during the acute stage. The most common first-line therapy was IVMP, which was administered to 11 patients. Dexamethasone and PSL were used in one episode each. All patients showed improvement during the acute stage, and 10 cases did not have sequelae. Of these 10 cases, nine exhibited a significant response to steroids during the acute stage, with five relying solely on steroids and four using a combination of steroids and other immunosuppressive medications. For follow-up treatment, six cases were treated with steroids alone, while three received no further treatment. In contrast, three patients demonstrated residual sequelae during follow-up. During the acute phase, one of these patients was treated with steroids alone, while the remaining two were treated with a combination of steroids and other immunosuppressive agents. The response to the acute-phase treatment was either slow or partial in all three cases. Relapses occurred in five cases, one of which (Case 7) occurred during steroid tapering and after the discontinuation of steroid use in another two (Cases 1 and 11).

## 4 Discussion

We identified a case of encephalitis that tested positive for both anti-NMDAR and anti-MOG antibodies and that responded well to steroid therapy during the acute stage. At the time of IVMP initiation, we suspected autoimmune encephalitis, specifically anti-NMDAR encephalitis, due to the presence of psychiatric symptoms and the absence of obvious lesions on imaging. Contrary to our expectations, the patient responded rapidly and well to steroid-only treatment, with some of his symptoms resolving by the next day. He was discharged a month later without any lasting effects after having received two IVMP courses. It was later discovered that the patient was positive not only for anti-NMDAR antibodies but also for anti-MOG antibodies.

Previous studies have suggested that both antibodies contribute to the development of overlapping encephalitis (10, 11). However, another report suggested that one of the antibodies might be a bystander in the process (19). We propose that the co-occurrence of anti-NMDAR and anti-MOG antibodies in autoimmune encephalitis represents a novel form of the condition that diverges from anti-NMDAR encephalitis or MOGAD alone and that each antibody has its own unique pathological implications, as discussed below. Anti-NMDAR IgG antibodies exhibit a pathogenic nature (25). Given that patients with overlapping encephalitis frequently exhibit symptoms similar to anti-NMDAR encephalitis, including psychiatric symptoms and seizures (10, 12, 13), we believe that anti-NMDAR antibodies also have pathological significance in overlapping encephalitis. Furthermore, our study revealed that psychiatric symptoms and seizures were common. According to the recent diagnostic criteria for MOGAD, antibody titers are believed to be important for the pathological significance of anti-MOG antibodies (5). Although anti-MOG antibody titers can vary among patients with overlapping encephalitis, the response to steroid treatment and high relapse rates in this condition are challenging to attribute to the clinical phenotype exhibited by anti-NMDAR antibodies and are similar to the clinical features of MOGAD (10, 20, 26). Thus, we believe that the pathological significance of anti-MOG antibodies in overlapping encephalitis is substantial.

Previous studies have presented potential mechanisms for the co-occurrence of anti-NMDAR and anti-MOG antibodies; however, the underlying reasons for their coexistence remain unclear. One key observation that may provide insight into the presence of these antibodies is that oligodendrocytes display functional NMDARs on their surfaces (27, 28). Damage to oligodendrocytes can potentially expose the antigens of both NMDAR and MOG proteins, leading to production of both antibodies (26, 29). Additionally, viral infections may play a role in the co-occurrence of these antibodies (19, 21, 30). Immune reconstitution during immunotherapy has also been suggested as a possible reason for the presence of both types of antibodies (31).

Several case reports on overlapping syndromes have demonstrated significant improvements after steroid treatment. However, studies exploring the effects of steroid therapy across multiple cases are lacking. In a study of five patients with overlapping syndromes, by Fan et al., high-dose steroids and IVIG were used as treatment during the acute phase, which resulted in a significant improvement in the modified Rankin Scale (mRS) score after a median of 18 months (32). Hou et al. used methylprednisolone and IVIG as treatment during the acute phase in seven children with this overlapping syndrome, and in a follow-up study of these patients approximately 1–2 years later, all patients had an mRS score of less than 1 (33).

In this study, we reviewed the efficacy of steroid therapy in 13 patients with encephalitis with coexisting anti-NMDAR and anti-MOG antibodies. Notably, all patients showed improvement during the acute-phase steroid treatment. Furthermore, 76.9% of the patients had favorable outcomes, with no long-term effects, and most experienced rapid or major recovery during the acute stage. Some patients showed improvement within a few days after treatment, whereas others who were discharged recovered within a month or were asymptomatic at the time of discharge. These results suggest that patients with overlapping encephalitis who respond well to steroid treatment during the acute stage may have a positive prognosis. In contrast, three patients remained symptomatic. Interestingly, their improvement after the initial steroid treatment was slow or limited. Despite the fact that their sequelae were generally mild, and the observation periods were short, it is possible that patients who do not show significant early improvement with steroid treatment may have long-term consequences. However, further studies are required to confirm this hypothesis.

The reason for the response of patients with anti-NMDAR and anti-MOG antibody overlapping encephalitis to steroid therapy is not yet completely understood; however, it may be explained by the involvement of anti-MOG antibodies in the pathogenesis of the disease. MOGAD has been reported to exhibit features of responding to steroids during acute phase treatment and relapse upon tapering or discontinuing steroid treatment (6, 34). In this study, many patients responded well to steroid treatment during the acute phase, while three of the five relapse episodes occurred during tapering or after discontinuation of steroid treatment. Another hypothesis for the high treatment response to steroids in overlapping syndromes is the low titers of anti-NMDAR antibodies. All cases in which anti-NMDAR antibody titers were measured in this study had low titers. A literature review of FLAIR hyperintense lesions in MOG-associated encephalitis with seizures with anti-NMDAR antibodies, reported by Yang et al., also found low levels of anti-NMDAR antibodies in the CSF, with titers ranging from 1:1 to 1:32 (22). Other studies have demonstrated that anti-NMDAR antibody titers are associated with symptom severity and prognosis and that patients with low levels of these antibodies typically exhibit milder symptoms and more favorable outcomes (35–38).

Our study has several limitations. First, the sample size was too small to provide a clear picture of the response to steroid treatment, due to the rarity of the coexistence of anti-NMDAR and anti-MOG antibodies and the limited number of cases with coexisting antibodies prior to treatment. Second, the information available on treatment response varied between episodes, with the speed and magnitude of recovery being difficult to quantify in some cases. Moreover, the duration of follow-up differed, with some papers describing follow-up periods of less than 6 months. Finally, there were episodes in which immunotherapy was used in combination with steroids, making it difficult to determine the pure effect of steroid therapy because the combination may have altered the therapeutic response.

In conclusion, our patient was diagnosed with anti-NMDAR and anti-MOG antibody overlapping encephalitis due to the presence of anti-NMDAR antibodies and high levels of anti-MOG antibodies, along with symptoms indicative of anti-NMDAR encephalitis and significant improvement after steroid treatment, which suggests that both antibodies are involved in the disease process. Our study suggest that steroid therapy is beneficial for acute phase treatment of anti-NMDAR and anti-MOG antibody overlapping encephalitis. Patients who showed rapid or significant improvements with steroid therapy tended to have a good prognosis. This approach may help avoid additional immunotherapy and ensure careful follow-up. Therefore, based on their history and symptoms, we recommend measuring anti-MOG antibodies in patients who respond well to steroids, even if anti-NMDAR encephalitis is suspected. Further prospective studies with a large number of cases and detailed documentation are needed to provide proper guidance for acute phase and follow-up treatments of overlapping encephalitis.

## Data availability statement

The original contributions presented in the study are included in the article/supplementary material. Further inquiries can be directed to the corresponding author.

## Ethics statement

Ethical approval was not required for the study involving humans in accordance with the local legislation and institutional requirements. Written informed consent to participate in this study was not required from the participants or the participants’ legal guardians/next of kin in accordance with the national legislation and the institutional requirements. Written informed consent was obtained from the individual(s) for the publication of any potentially identifiable images or data included in this article.

## Author contributions

HK: Writing – original draft, Writing – review & editing, Methodology, Validation. YT: Writing – review & editing. JN: Writing – review & editing. KY: Writing – review & editing. NT: Writing – review & editing. SH: Data curation, Writing – review & editing. TK: Writing – review & editing. KK: Investigation, Writing – review & editing. NM: Supervision, Writing – review & editing.
